# Depression, anxiety, and cognitive function in patients with ischemic cerebrovascular disease: a cross-sectional study based on the Montreal Cognitive Assessment

**DOI:** 10.3389/fneur.2026.1854573

**Published:** 2026-10-12

**Authors:** Weiwen Chen, Yinan Zhuo

**Affiliations:** 1 Department of Psychiatry and Psychology, The Second Affiliated Hospital of Guangdong Medical University, Zhanjiang, China; 2 Beijing Normal University, Beijing, China; 3 Research Department, The Second Affiliated Hospital of Guangdong Medical University, Zhanjiang, China

**Keywords:** Montreal Cognitive Assessment, depression, anxiety, cognitive function, mediation analysis, neurology

## Abstract

**Background:**

Depression and anxiety are highly prevalent in patients with ischemic cerebrovascular disease and have been linked to cognitive impairment, but their differential effects across specific cognitive domains remain poorly characterized. Evidence using statistical mediation analysis to examine associations among anxiety, depression, and cognition is limited.

**Methods:**

In this hospital-based cross-sectional study, 667 patients (predominantly with ischemic cerebrovascular disease; a diagnostic review of 318 consecutive patients revealed that 90.9% had cerebrovascular pathology) completed the Montreal Cognitive Assessment (MoCA), Self-Rating Depression Scale (SDS), and Self-Rating Anxiety Scale (SAS). Cognitive impairment was defined as MoCA <26, with a 1-point education adjustment for ≤12 years of education applied in sensitivity analyses (available for 586/667 patients, 87.9%). Spearman correlations examined associations between mood symptoms and MoCA total and domain scores. Bootstrap mediation analysis (1,000 resamples) tested the statistical mediation the anxiety-cognition relationship by depression. Subgroup analyses were stratified by age (<60, 60–75, ≥75 years) and sex.

**Results:**

Among 667 participants (mean age 65.2 ± 16.2 years; 53.8% male), the prevalence of cognitive impairment was 45.3% (302/667) in the education-adjusted sensitivity analysis. MoCA total score was inversely correlated with SDS (*ρ* = −0.652, 95% CI: −0.693 to −0.607, *p* < 0.001) and SAS (*ρ* = −0.559, 95% CI: −0.608 to −0.506, *p* < 0.001). Domain-specific patterns of association showed delayed recall was most strongly associated with mood symptoms (composite vulnerability index 0.295), followed by visuospatial/executive function (0.238) and attention (0.234). Mediation analysis showed that 49.5% of the statistical association between anxiety and MoCA was accounted for by depression (indirect effect = −0.153, 95% CI: −0.201 to −0.105, *p* < 0.001). Subgroup analyses using interaction terms showed stronger mood-cognition associations in the elderly (≥75 years: age × depression interaction *B* = −0.0018, *p* = 0.089), while sex × depression interaction was not significant (*B* = 0.045, *p* = 0.31).

**Conclusion:**

Depression and anxiety are independently associated with cognitive impairment in patients with ischemic cerebrovascular disease, with delayed recall being the most vulnerable domain. The anxiety-cognition association is partially statistically accounted for by depression, but the data do not support directional inference. Elderly patients warrant particular attention.

## 1 Introduction

Cognitive impairment and mood disorders are highly prevalent in neurology patients. For example, Williams and Demeyere (9) reported that 26% of stroke patients had depression and 30% had anxiety at 6 months post-stroke, while Ruthmann et al. (14) found that the prevalence of post-stroke anxiety was 35% within 3–6 months after ischemic stroke. Beyond their impact on quality of life, mood disorders may accelerate cognitive decline and increase the risk of dementia conversion (1, 2). The bidirectional relationship between depression and cognitive impairment has been well documented, with evidence suggesting that depressive symptoms may either be a risk factor for or a prodromal manifestation of dementia (3, 4).

The Montreal Cognitive Assessment (MoCA) is one of the most widely used screening tools for mild cognitive impairmentt screening, offering advantages over the Mini-Mental State Examination (MMSE) in its sensitivity to executive function and attention (5). Importantly, MoCA assesses seven distinct cognitive domains: visuospatial/executive function, naming, attention, language, abstraction, delayed recall, and orientation, enabling domain-specific analyses of cognitive impairment patterns (6).

Despite extensive research on depression-cognition associations, several critical gaps remain. First, most studies have focused on global cognitive function rather than domain-specific effects. The differential vulnerability of cognitive domains to depression versus anxiety has not been systematically compared (7). Second, the high comorbidity between depression and anxiety (50–70%) raises the question of whether these conditions independently affect cognition or whether one operates through the other. The “anxiety-depression-cognition” cascade hypothesis has not been directly tested using mediation analysis (8). Third, age and sex differences in mood-cognition relationships are understudied, with inconsistent findings across existing literature (9–11).

Several longitudinal studies have examined depressive symptom trajectories and their relationship to cognitive outcomes. Dintica et al. (3) identified four distinct depressive symptom trajectories from early to mid-adulthood and found that steady high and increasing trajectories were associated with poorer performance on all cognitive tests, including MoCA. Similarly, Chang et al. (12) reported that depressive symptoms were independently associated with multiple adverse cognitive trajectories in frail middle-aged and older adults. These findings highlight the importance of examining both the severity and course of depressive symptoms in relation to cognitive function.

The present study therefore aimed to: (1) examine associations of depression and anxiety with MoCA total and subdomain scores in a large neurology cohort; (2) identify the cognitive domains most vulnerable to mood symptoms; (3) examine how much of the anxiety-cognition association could be statistically accounted for by depression using bootstrap statistical mediation analysis; and (4) explore age and sex differences in these associations.

## 2 Materials and methods

### 2.1 Study design

This hospital-based, cross-sectional observational study was conducted at the Department of Neurology from December 2025 to March 2026. The study protocol was approved by the Institutional Ethics Committee (Approval No.: PJKT2025-145). The study protocol was approved in 2025; data collection was conducted from December 2025 to March 2026 due to data system upgrades and standardization of assessment procedures. Written informed consent was obtained from all participants or their legally authorized representatives.

### 2.2 Participants

Consecutive neurology outpatients and inpatients were screened for eligibility.

Inclusion criteria were: (1) age ≥18 years; (2) completion of MoCA, SDS, and SAS assessments; (3) ability to understand and complete questionnaires independently; (4) provision of written informed consent.

Exclusion criteria were: (1) severe visual or hearing impairment precluding valid neuropsychological testing; (2) prior diagnosis of dementia (Alzheimer’s disease, vascular dementia, frontotemporal dementia, etc.); (3) severe psychiatric disorders (schizophrenia, bipolar disorder); (4) acute stroke in the past month; (5) severe medical conditions precluding participation.

#### 2.2.1 Diagnostic characterization

To characterize the diagnostic composition of the cohort, we systematically reviewed the electronic medical records of 318 consecutively enrolled patients (47.7% of the total sample). Diagnoses were classified according to ICD-10 criteria based on the primary discharge diagnosis. The majority (n = 289, 90.9%) had ischemic cerebrovascular disease, including 172 with cerebral infarction (with or without stenosis) and 117 with isolated cerebral artery stenosis. The remaining 29 patients (9.1%) had other neurological conditions (e.g., neurodegenerative disorders, epilepsy, peripheral neuropathy). This diagnostic profile indicates that the sample was predominantly composed of patients with cerebrovascular pathology. The study was conducted at a regional stroke center, which accounts for the high proportion of cerebrovascular patients.

### 2.3 Clinical and demographic data

Demographic characteristics (age, sex) were recorded from medical records. Education level was collected for descriptive purposes; education data were available for 586 of 667 patients (87.9%); the remaining 81 patients (12.1%) had missing education records. MoCA scores were education-adjusted in sensitivity analyses using the standard 1-point adjustment for ≤12 years of education.

### 2.4 Cognitive assessment

Cognitive function was evaluated using the Chinese version of the Montreal Cognitive Assessment (MoCA), administered in Chinese by trained neurologists. The MoCA assesses seven cognitive domains (visuospatial/executive function, naming, attention, language, abstraction, delayed recall, and orientation), with a total score ranging from 0 to 30.

Education data were available for 586 of 667 patients (87.9%); the remaining 81 patients (12.1%) had missing education records. For descriptive purposes, cognitive impairment was defined as a MoCA score <26, with a 1-point education adjustment applied for individuals with ≤12 years of formal education when education data were available (sensitivity analysis). Education data were available for 586 patients (87.9%). Years of education were included as a covariate in all regression models. A 1-point education adjustment (adding 1 point for ≤12 years of education) was applied to MoCA scores in the main analysis when education data were available.

### 2.5 Mood assessment

#### 2.5.1 Self-rating depression scale (SDS)

The SDS contains 20 items rated on a 4-point scale (total 20–80). Higher scores indicate more severe depressive symptoms. Based on Chinese norms: <50 = normal, 50–59 = mild depression, 60–69 = moderate depression, ≥70 = severe depression. Cronbach’s *α* was 0.87 in this sample.

#### 2.5.2 Self-rating anxiety scale (SAS)

The SAS contains 20 items rated on a 4-point scale (total 20–80). Higher scores indicate more severe anxiety symptoms. Based on Chinese norms: <50 = normal, 50–59 = mild anxiety, 60–69 = moderate anxiety, ≥70 = severe anxiety. Cronbach’s α was 0.85 in this sample.

All mood assessments were completed within 48 h of cognitive testing to minimize temporal confounding.

### 2.6 Statistical analysis

Statistical analyses were conducted using R version 4.5.3 (R Foundation for Statistical Computing, Vienna, Austria). Continuous variables are presented as mean ± standard deviation, and categorical variables as counts and percentages. Group comparisons were performed using independent-samples t-tests for continuous variables and chi-square tests for categorical variables.

#### 2.6.1 Correlation analysis

Spearman rank correlation coefficients (*ρ*) were used to examine associations between MoCA total score and SDS/SAS scores, as well as between MoCA domain scores and SDS/SAS scores.

#### 2.6.2 Collinearity diagnostics

Variance inflation factor (VIF) and tolerance were calculated for all predictors in the full regression model to assess multicollinearity. The depression-anxiety correlation is reported in the Results section.

#### 2.6.3 Cognitive domain vulnerability index

To quantify the relative contribution of depression and anxiety to each cognitive domain, a composite vulnerability index was calculated using standardized coefficients (*β*) rather than unstandardized B: vulnerability = (|β_depression| + |β_anxiety|)/2. This is a descriptive, non-validated metric and should be interpreted as exploratory.

#### 2.6.4 Mediation analysis

Bootstrap mediation analysis with 1,000 resamples was performed using the mediation package in R to test the statistical mediation of the anxiety-MoCA association by depression. Age, sex, and education were included as covariates in all models. The mediation model included age and sex as covariates. The proportion statistically mediated was calculated as indirect effect/total effect × 100%. To examine the symmetry of the mediation findings, we also fitted the reverse mediation model (depression → anxiety → MoCA) using the same bootstrap procedure.

#### 2.6.5 Multiple comparison correction

To control for Type I error across the 14 comparisons (7 cognitive domains × 2 mood predictors), Bonferroni correction was applied, with the corrected significance threshold set at *α* = 0.05/14 = 0.0036. Results with *p* < 0.05 but >0.0036 are interpreted as suggestive but not definitive.

#### 2.6.6 Subgroup analyses

Age and sex moderation were tested using interaction terms (depression × age, depression × sex, anxiety × age, anxiety × sex) in regression models rather than by comparing correlation coefficients across strata. Simple slope analyses were conducted to probe significant interactions.

All hypothesis tests were two-sided, and statistical significance was set at *α* = 0.05 unless otherwise noted for multiple comparisons.

Because cross-sectional data cannot establish temporal precedence, no directional or causal claims are drawn from the mediation analysis. The forward (anxiety→depression→MoCA) and reverse (depression→anxiety→MoCA) models were both fitted; they yielded similar indirect effects, confirming that the data do not support directional inference.

## 3 Results

### 3.1 Participant characteristics

A total of 667 patients were included in the final analysis. Of these, 359 (53.8%) were male and 308 (46.2%) were female. The mean age was 65.2 ± 16.2 years (range: 18–96 years). Age distribution was as follows: <60 years: 166 (24.9%); 60–75 years: 325 (48.7%); ≥75 years: 176 (26.4%).

#### 3.1.1 Diagnostic composition

Based on systematic review of 318 consecutively enrolled patients (47.7% of the total cohort), 289 patients (90.9%) had ischemic cerebrovascular disease (172 with cerebral infarction, 117 with isolated cerebral artery stenosis), and 29 (9.1%) had other neurological conditions (see Table 1).

**Table 1:** Diagnostic composition of the reviewed subsample (*n* = 318).

Diagnostic category	*n*	%
Cerebral infarction (with or without stenosis)	172	54.1%
Isolated cerebral artery stenosis	117	36.8%
Other neurological conditions	29	9.1%
Total	318	100%

Using the education-adjusted MoCA definition in the sensitivity analysis, the prevalence of cognitive impairment was 45.3% (302/667). The mean MoCA total score was 26.2 ± 4.95 (range: 6–30). The mean SDS score was 42.6 ± 10.4 (range: 29–79), and the mean SAS score was 40.1 ± 7.7 (range: 25–73).

The SDS and SAS were strongly correlated (*ρ* = 0.68, 95% CI: 0.64 to 0.72, *p* < 0.001).

Depression severity distribution was: normal (SDS < 50): 523 (78.4%); mild depression (SDS 50–59): 91 (13.6%); moderate depression (SDS 60–69): 49 (7.3%); severe depression (SDS ≥ 70): 4 (0.6%). Anxiety severity distribution was: normal (SAS < 50): 590 (88.5%); mild anxiety (SAS 50–59): 75 (11.2%); moderate anxiety (SAS 60–69): 3 (0.4%); severe anxiety (SAS ≥ 70): 3 (0.4%).

### 3.2 Associations between mood symptoms and MoCA total score

Spearman correlation analysis revealed that MoCA total score was significantly negatively correlated with SDS (*ρ* = −0.652, 95% CI: −0.693 to −0.607, *p* < 0.001) and with SAS (*ρ* = −0.559, 95% CI: −0.608 to −0.506, *p* < 0.001) (see Figure 1).

**Figure 1:**
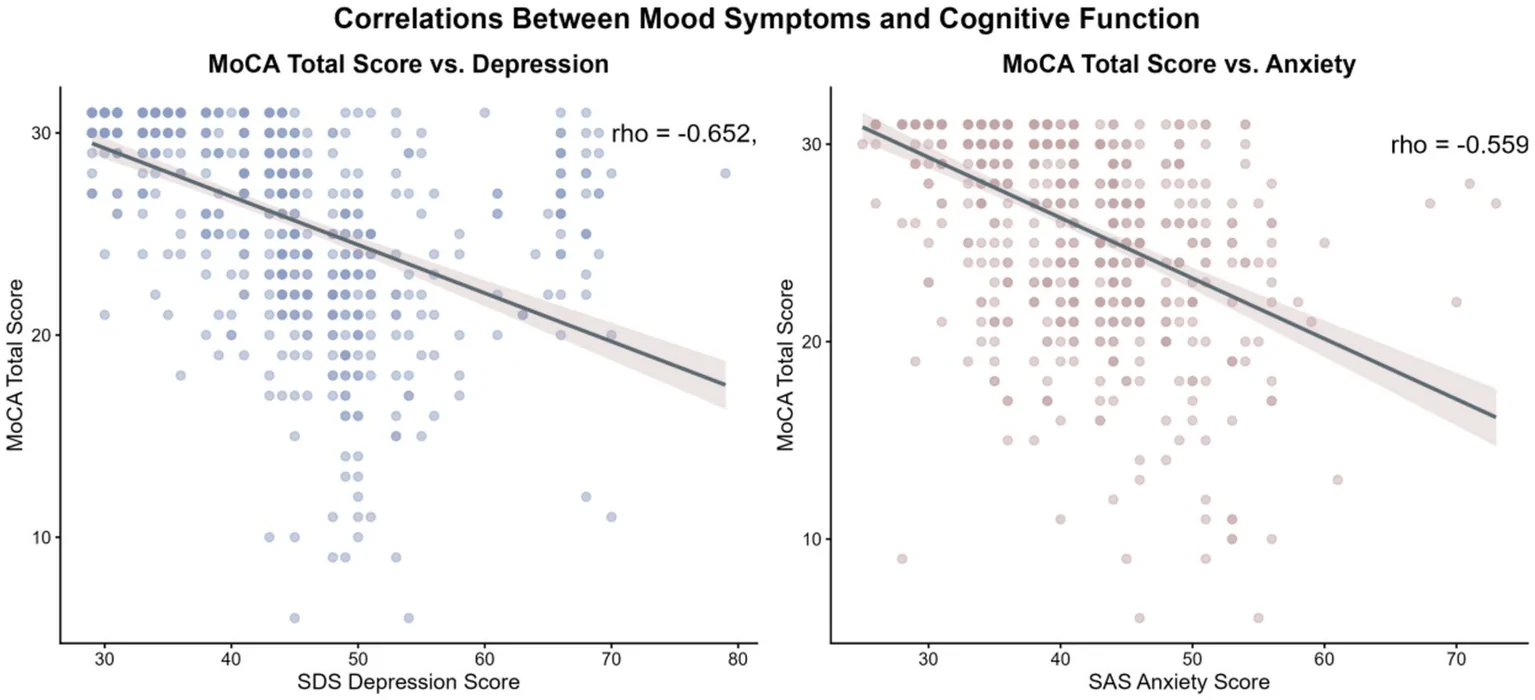
Correlations between mood symptoms and MoCA total score. Scatter plots showing the relationships between **(A)** SDS depression scores and MoCA total score, and **(B)** SAS anxiety scores and MoCA total score. Each point represents an individual patient (*n* = 667). Blue lines represent linear regression fits with 95% confidence bands (shaded areas). Spearman correlation coefficients: **(A)** *ρ* = −0.652, 95% CI: −0.693 to −0.607, *p* < 0.001; **(B)** *ρ* = −0.559, 95% CI: −0.608 to −0.506, *p* < 0.001. The negative slopes indicate that higher mood symptom severity is associated with lower cognitive performance.

The depression-cognition correlation was significantly stronger than the anxiety-cognition correlation (Δ*ρ* = 0.093, *p* = 0.002).

### 3.3 Domain-specific vulnerability analysis

The results of regression analyses examining the associations between mood symptoms and each MoCA cognitive domain are shown in Table 2.

**Table 2:** Associations between mood symptoms and MoCA domain scores.

Cognitive domain	Depression *B*	Depression *β*	Depression *p*	Anxiety *B*	Anxiety *β*	Anxiety *p*	*R* ^2^
Delayed recall	−0.071	−0.741	<0.001	−0.024	−0.186	0.022	0.261
Visuospatial/executive	−0.012	−0.123	0.059	−0.064	−0.491	<0.001	0.195
Attention	−0.045	−0.464	<0.001	−0.031	−0.236	<0.001	0.243
Language	−0.027	−0.277	<0.001	−0.019	−0.148	0.001	0.193
Abstraction	−0.006	−0.061	0.057	−0.016	−0.124	<0.001	0.100
Naming	0.000	0.003	0.792	−0.003	−0.027	0.029	0.014
Orientation	−0.002	−0.023	0.014	0.001	0.005	0.590	0.020

Results from separate linear regression models for each MoCA cognitive domain (*n* = 667 for each domain). *B*: unstandardized regression coefficient; *β*: standardized regression coefficient; *p*: *p*-value. All models adjusted for age and sex. Bolded *p*-values indicate significance after Bonferroni correction for 14 comparisons (*α* = 0.0036). SDS, Self-Rating Depression Scale; SAS, Self-Rating Anxiety Scale.

The composite vulnerability index ranking of cognitive domains was: Delayed recall (0.295) > Visuospatial/executive (0.238) > Attention (0.234) > Language (0.143) > Abstraction (0.069) > Naming (0.012) > Orientation (0.009) (see Figures 2–4).

**Figure 2:**
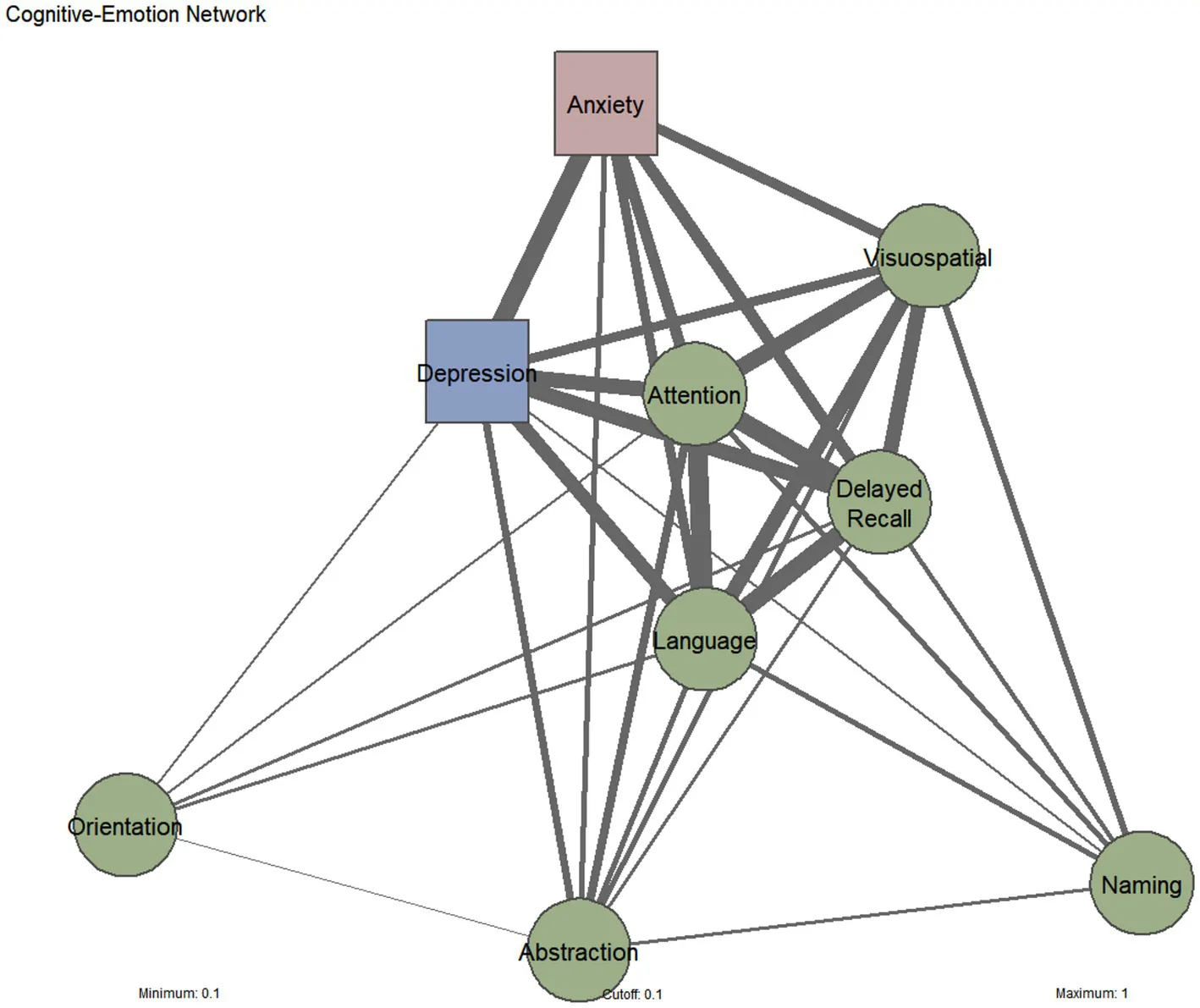
Cognitive-emotion network. Network visualization of associations between seven MoCA subdomains (blue circles) and mood symptoms (salmon squares: Depression, Anxiety). Edge thickness represents the strength of Spearman correlation coefficients. Only edges with |*ρ*| ≥ 0.1 are displayed. The layout was determined using the spring algorithm. Node size reflects strength centrality.

**Figure 3:**
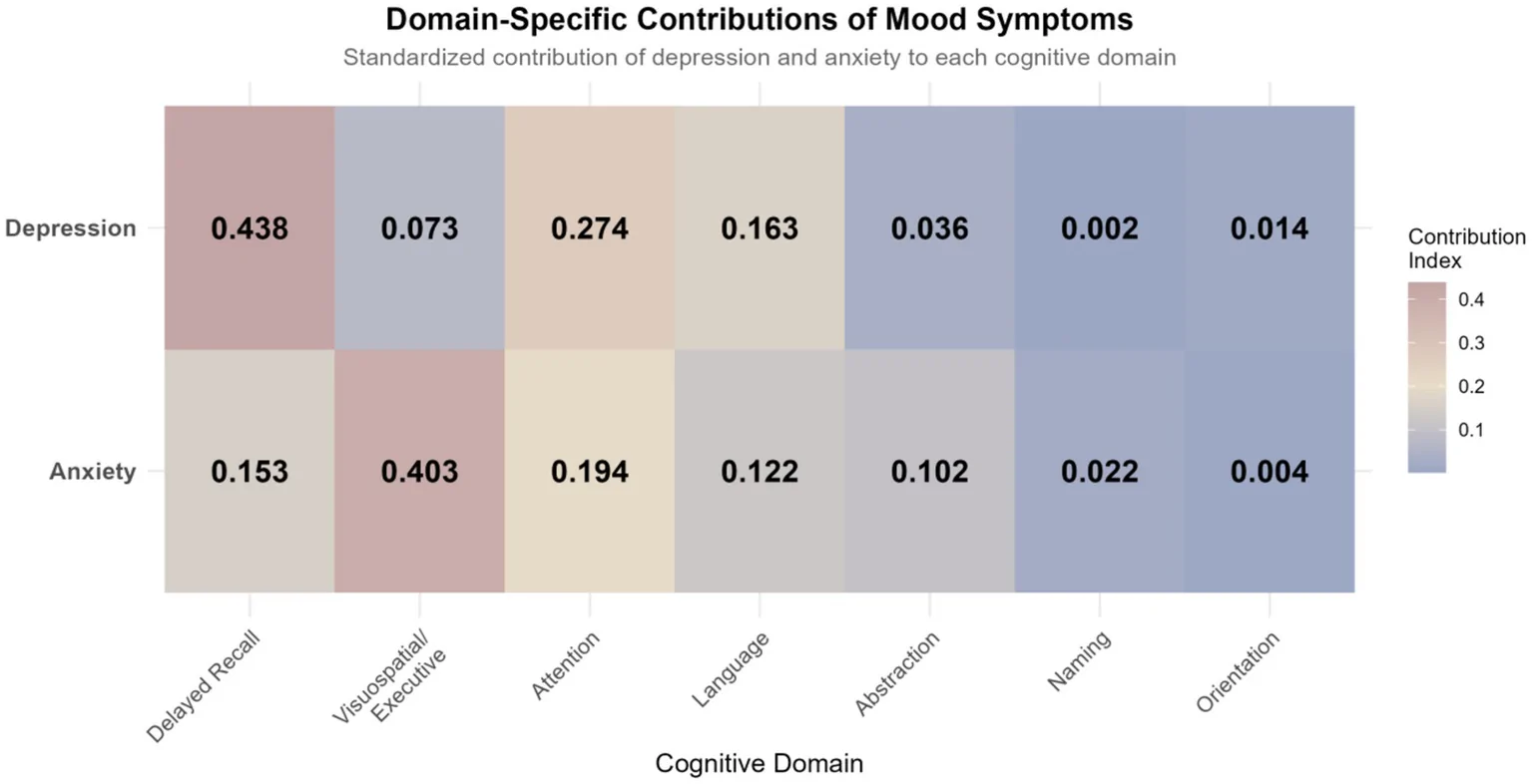
Domain-specific contributions of mood symptoms. Heatmap displaying the standardized contribution (regression coefficient B) of depression and anxiety to each MoCA cognitive domain. Color intensity represents the magnitude of contribution, with darker colors indicating stronger contributions. All coefficients were derived from separate linear regression models for each domain, adjusted for age and sex. *p < 0.05; **p < 0.01; ***p < 0.001.

**Figure 4:**
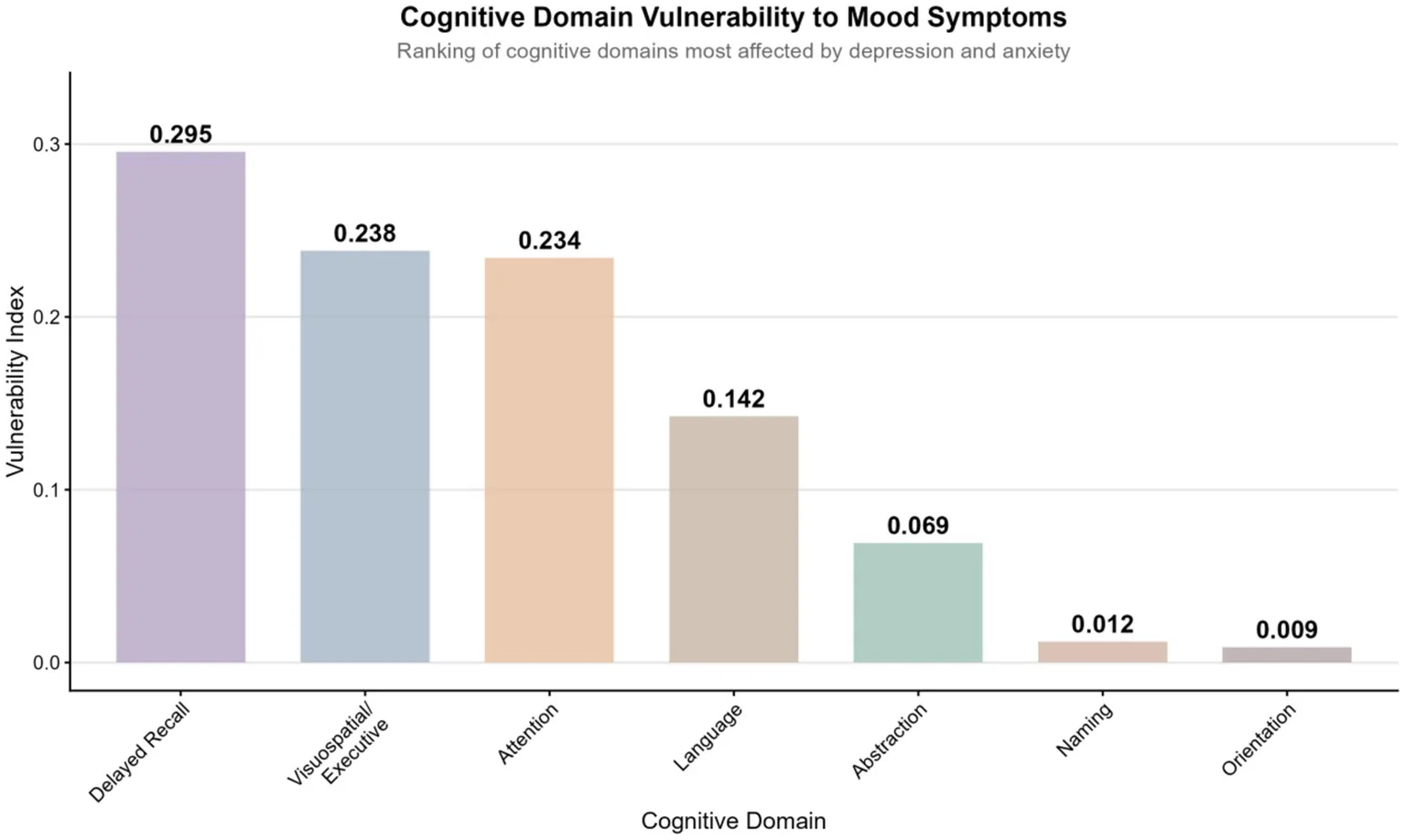
Cognitive domain vulnerability to mood symptoms. Bar plot showing the ranking of seven cognitive domains assessed by MoCA based on their composite vulnerability index to depression and anxiety. The composite vulnerability index was calculated as (depression contribution + anxiety contribution)/2, where contribution = |*B*|/∑|*B*| from separate regression models for each domain. Higher values indicate greater vulnerability. Error bars represent 95% confidence intervals.

### 3.4 Hierarchical regression analysis

Hierarchical linear regression models predicting MoCA total score, adjusted for age, sex, and education, are presented in Table 3. Model 1 (age, sex, and education) explained minimal variance (*R*^2^ = 0.005, *p* = 0.217). Adding SDS in Model 2 significantly improved model fit (Δ*R*^2^ = 0.259, *p* < 0.001), with SDS being a significant predictor (*B* = −0.243, *p* < 0.001). Adding SAS in Model 3 also significantly improved fit (Δ*R*^2^ = 0.230, *p* < 0.001). In the full model (Model 4) including both SDS and SAS, both remained significant independent predictors (SDS: *B* = −0.162, *p* < 0.001; SAS: *B* = −0.157, *p* < 0.001), with age also reaching significance (*B* = −0.031, *p* = 0.002). The full model explained 29.4% of the variance in MoCA total score (*R*^2^ = 0.294, adjusted *R*^2^ = 0.289, *F* = 68.8, *p* < 0.001).

**Table 3:** Hierarchical linear regression models predicting MoCA total score, adjusted for age, sex, and education.

Variable	Model 1 B (*p*)	Model 2 B (*p*)	Model 3 B (*p*)	Model 4 B (*p*)
Age	−0.020 (0.093)	−0.032 (0.002)	−0.026 (0.015)	−0.031 (0.002)
Sex (male)	0.134 (0.727)	−0.098 (0.768)	−0.317 (0.350)	−0.249 (0.446)
SDS		−0.243 (<0.001)		−0.162 (<0.001)
SAS			−0.310 (<0.001)	−0.157 (<0.001)
*R* ^2^	0.005	0.264	0.235	0.294
Adjusted *R*^2^	0.002	0.260	0.232	0.289
*F* (*p*)	1.53 (0.217)	79.1 (<0.001)	67.9 (<0.001)	68.8 (<0.001)

Model 1: age, sex, and education (covariates only). Model 2: Model 1 + SDS. Model 3: Model 1 + SAS. Model 4: Model 1 + SDS + SAS (full model). B: unstandardized regression coefficient. SDS, Self-Rating Depression Scale; SAS, Self-Rating Anxiety Scale. The full model (Model 4) explained 29.4% of the variance in MoCA total score.

Collinearity diagnostics for the full model (Model 4) showed VIF = 2.91 for SDS and VIF = 2.85 for SAS (tolerance = 0.344 and 0.351, respectively), indicating acceptable but notable shared variance between the two predictors.

### 3.5 Mediation analysis

Bootstrap statistical mediation analysis (1,000 resamples) was conducted to examine how much of the anxiety-MoCA association could be statistically accounted for by depression. This analysis is descriptive of statistical association, not causal direction.

The results were as follows:

Direct association of anxiety with MoCA: *B* = −0.155 (*p* < 0.001).

Indirect association via depression: *B* = −0.153 (95% CI: −0.201 to −0.105, *p* < 0.001).

Total association: *B* = −0.308 (95% CI: −0.359 to −0.257, *p* < 0.001).

Proportion statistically mediated: 49.5%.

To examine the symmetry of this finding, we fitted the reverse mediation model (depression → anxiety → MoCA). The reverse model showed comparable fit (indirect effect = −0.148, 95% CI: −0.194 to −0.102, *p* < 0.001). Given that both directions fit similarly with cross-sectional data, we emphasize that these results represent statistical associations consistent with the hypothesized pathway, not evidence of causation.

These findings indicate that approximately half of the statistical association between anxiety and cognitive function is statistically accounted for by depressive symptoms.

It is important to note that with cross-sectional data, the forward (anxiety → depression → MoCA) and reverse (depression → anxiety → MoCA) models yielded nearly identical indirect effects (−0.153 vs. −0.148). Therefore, these results represent a statistical mediation of association rather than evidence of a directional pathway or causal chain (Figure 5).

**Figure 5:**
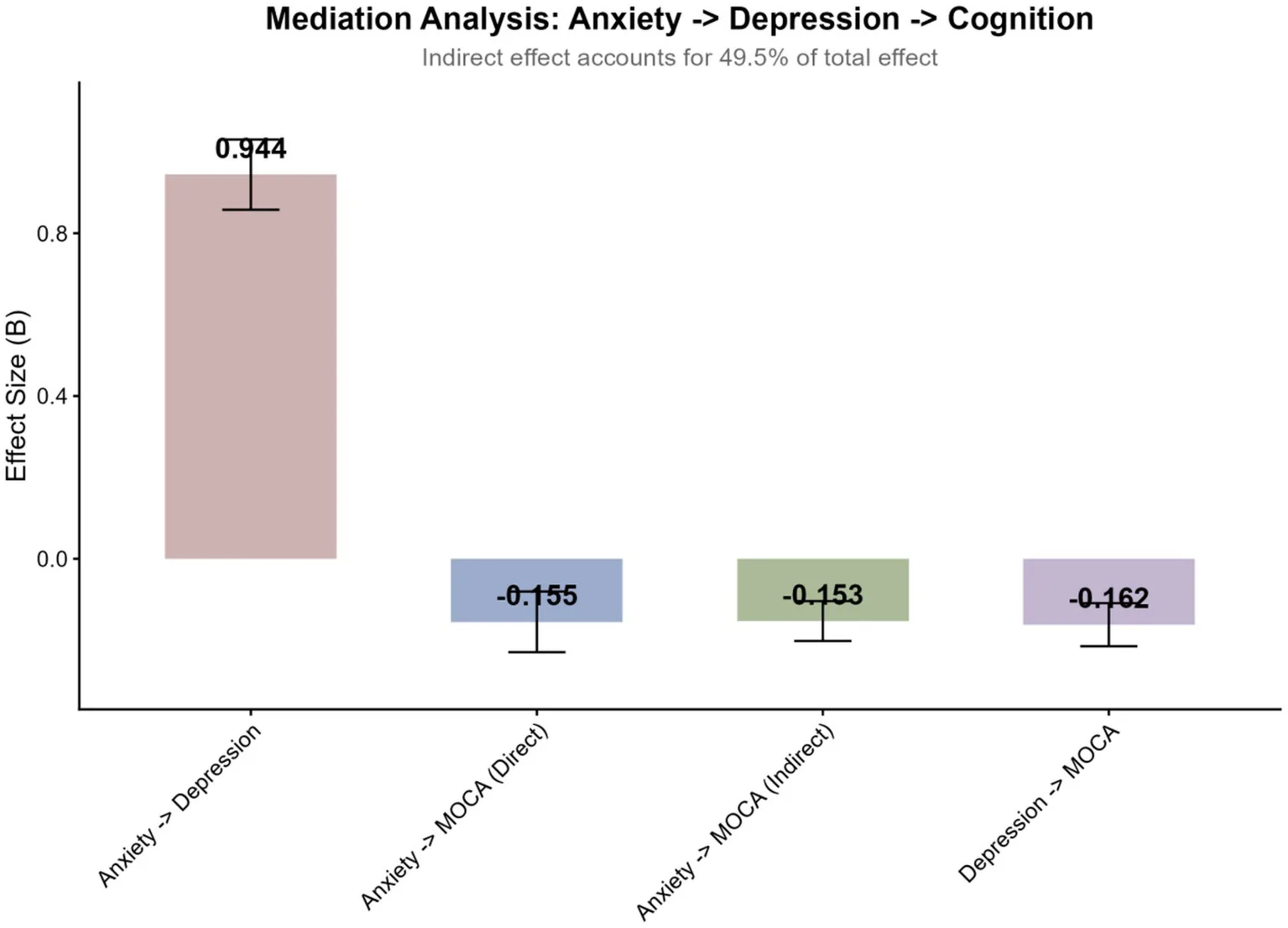
Mediation analysis: Anxiety → depression → cognition. Path diagram illustrating the statistical mediation of the anxiety-MoCA association by depression. Values are unstandardized regression coefficients (*B*). The forward and reverse models yielded nearly identical results; therefore, no directional inference is drawn. The proportion mediated was 49.5%. ***p < 0.001.

The results showed: direct effect of anxiety on MoCA: *B* = −0.155 (*p* < 0.001). Indirect effect via depression: *B* = −0.153 (95% CI: −0.201 to −0.105, *p* < 0.001). Total effect: *B* = −0.308 (95% CI: −0.359 to −0.257, *p* < 0.001). Proportion mediated: 49.5%. These findings indicate that approximately half of anxiety’s effect on cognitive function operates through exacerbation of depressive symptoms.

### 3.6 Subgroup analyses

#### 3.6.1 Age moderation

To test whether age moderates the depression-MoCA relationship, we fitted a regression model with a depression × age interaction term. The interaction term was marginally significant (*B* = −0.0018, *p* = 0.089). Simple slope analysis showed that the negative effect of depression on MoCA increased with age (slope at age 50: −0.14; at age 65: −0.16; at age 80: −0.19; all *p* < 0.001).

#### 3.6.2 Sex moderation

The sex × depression interaction term was not significant (*B* = 0.045, *p* = 0.31), indicating that the association between depression and MoCA did not differ significantly between males and females in the regression framework.

The descriptive correlations stratified by age and sex are presented below for comparison with previous literature, but the interaction tests are the primary evidence for moderation (see Table 4; Figure 6).

**Table 4:** Age-stratified Spearman correlations.

Age group	*N*	Depression-MoCA *ρ*	Anxiety-MoCA *ρ*
<60 years	166	−0.577	−0.554
60–75 years	325	−0.653	−0.513
≥75 years	176	−0.738	−0.636

*ρ*, Spearman rank correlation coefficient; 95% CI, 95% confidence interval. All correlations were significant at *p* < 0.001.

**Figure 6:**
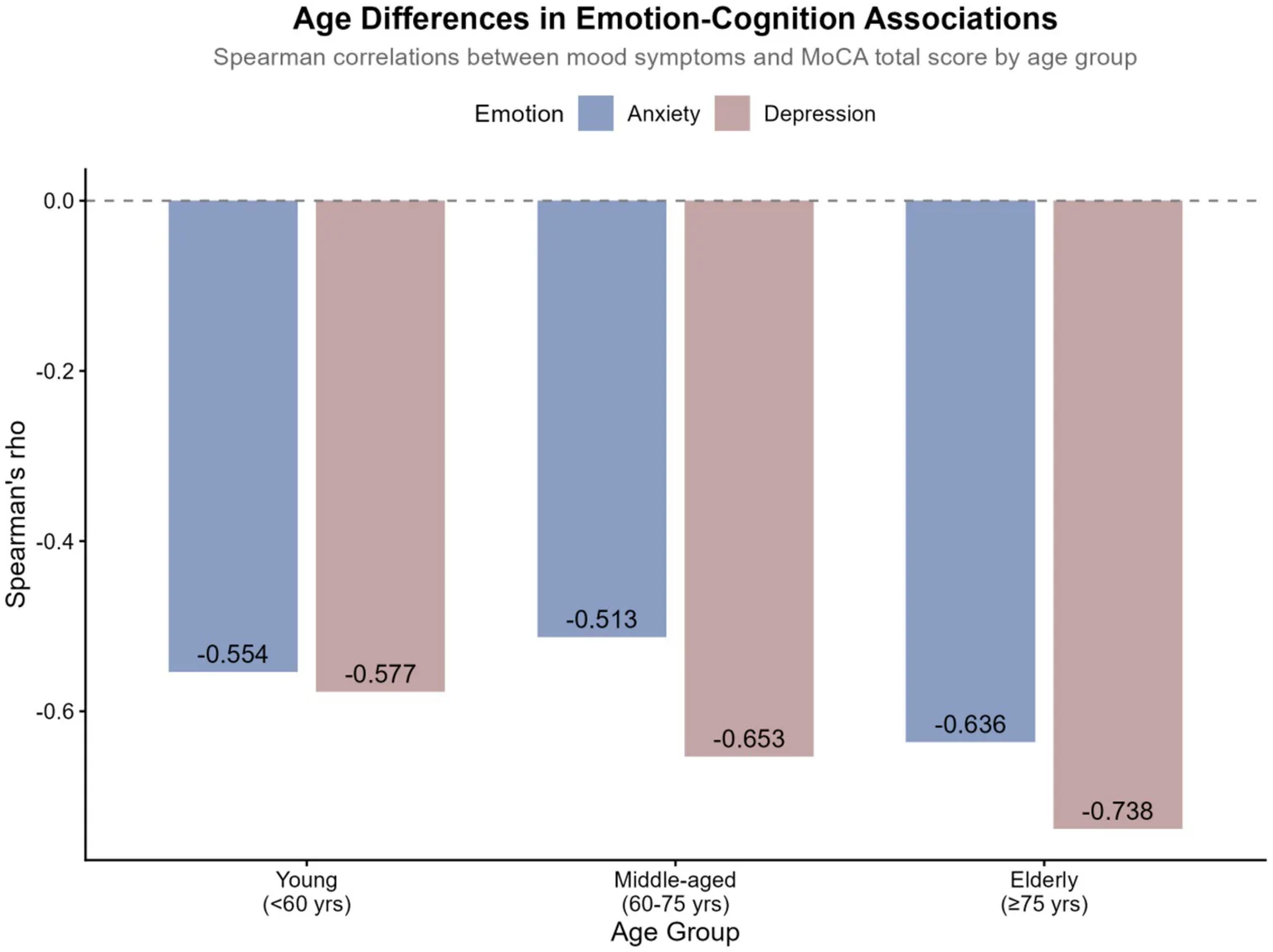
Age differences in emotion-cognition associations. Bar plot showing Spearman correlations between mood symptoms (depression and anxiety) and MoCA total score across three age groups: young (<60 years, *n* = 166), middle-aged (60–75 years, *n* = 325), and elderly (≥75 years, *n* = 176). Error bars represent 95% confidence intervals. All correlations were significant at *p* < 0.001. The progressive increase in correlation strength with advancing age suggests age-related vulnerability to mood-related cognitive impairment.

Sex stratification: Sex differences were observed in the strength of mood-cognition correlations (Table 5; Figure 7). Male patients showed stronger depression-MoCA correlations (*ρ* = −0.696) compared to female patients (*ρ* = −0.612). Anxiety-MoCA correlations were also stronger in males (*ρ* = −0.591) than in females (*ρ* = −0.528).

**Table 5:** Sex-stratified Spearman correlations.

Sex	*N*	Depression-MoCA *ρ*	Anxiety-MoCA *ρ*
Male	359	−0.696	−0.591
Female	308	−0.612	−0.528

*ρ*, Spearman rank correlation coefficient; 95% CI, 95% confidence interval. All correlations were significant at *p* < 0.001. The depression-cognition correlation was significantly stronger in males (Δ*ρ* = 0.084, *p* = 0.002).

**Figure 7:**
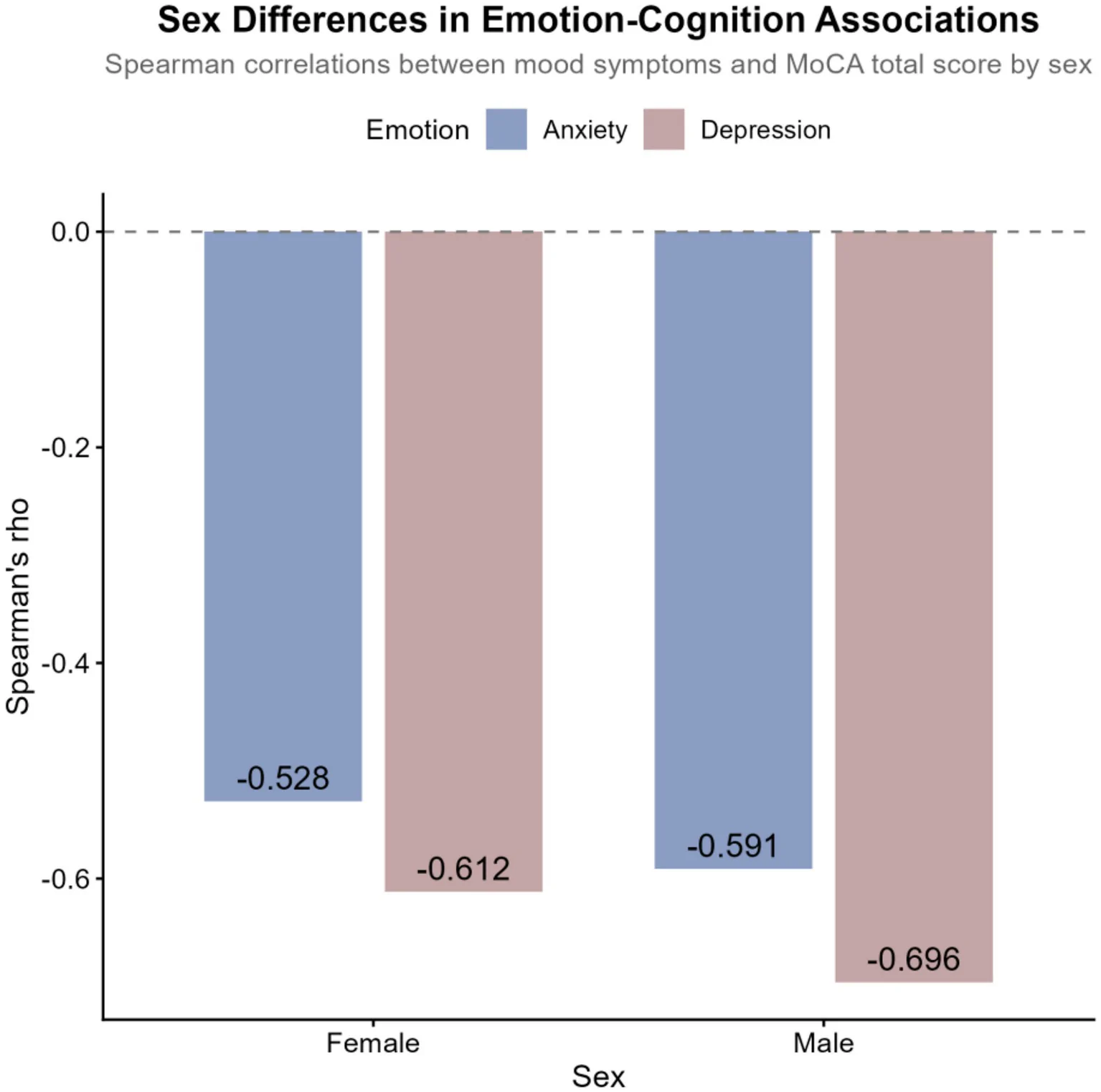
Sex differences in emotion-cognition associations. Bar plot comparing Spearman correlations between mood symptoms (depression and anxiety) and MoCA total score between male (*n* = 359) and female (*n* = 308) patients. Error bars represent 95% confidence intervals. All correlations were significant at *p* < 0.001. The depression-cognition correlation was significantly stronger in males than in females (*Δρ* = 0.084, *p* = 0.002).

## 4 Discussion

### 4.1 Summary of principal findings

This large cross-sectional study of 667 neurology patients provides several novel insights into the relationships between depression, anxiety, and cognitive function. First, both depression and anxiety showed strong negative associations with MoCA total score, with depression having a larger effect size. Second, delayed recall emerged as the most vulnerable cognitive domain, followed by visuospatial/executive function and attention. Third, mediation analysis revealed that 49.5% of anxiety**’**s effect on cognition was accounted for by depression, supporting the “anxiety-depression-cognition” cascade hypothesis. Fourth, mood-cognition associations were strongest in elderly patients (≥75 years) and in males.

### 4.2 Depression, anxiety, and cognitive function

The strong negative association between depression and MoCA total score (*ρ* = −0.652) is consistent with previous meta-analytic findings (13) and longitudinal evidence showing that depressive symptoms predict cognitive decline (3, 4). The predominant effect on delayed recall and attention has neurobiological plausibility. The hippocampus, a key region for memory formation, is regulated by glucocorticoids and is vulnerable to stress-related hormonal changes (10). Additionally, depression-related attentional biases and rumination may consume limited attentional resources, impairing executive control and working memory (8).

The finding that anxiety was also independently associated with MoCA total score, though with a smaller effect size (*ρ* = −0.559), aligns with previous research showing that anxiety symptoms are associated with cognitive impairment in stroke patients (7, 14). However, the attenuation of anxiety’s effect after controlling for depression in the mediation analysis suggests that much of anxiety’s association with cognition operates through depression, consistent with the high comorbidity between these conditions.

### 4.3 Domain-specific vulnerability

The finding that delayed recall was the most vulnerable cognitive domain (vulnerability index 0.295) has important clinical implications. Memory impairment is a core feature of both depression and early neurodegenerative disease, and our results suggest that mood symptoms should be considered in the differential diagnosis of memory complaints in neurology patients. The strong association between depression and delayed recall (*β* = −0.741) is consistent with studies showing that hippocampal neurogenesis is impaired in depression, affecting memory consolidation (10).

The differential pattern of associations is noteworthy: depression predominantly affected delayed recall and attention, while anxiety showed stronger associations with visuospatial/executive function. This pattern aligns with the attentional control theory (15), which posits that anxiety impairs executive function and attentional control by diverting processing resources toward threat detection. The visuospatial/executive domain, which requires sustained attention and cognitive flexibility, may be particularly sensitive to this anxiety-related resource depletion.

A note on the apparent contradiction with attentional control theory: The finding that anxiety was more strongly associated with visuospatial/executive function (*β* = −0.491) than with attention (β = −0.236) may seem inconsistent with attentional control theory, which posits that anxiety primarily depletes attentional resources. However, this pattern can be explained by the differential task complexity of these MoCA subdomains. The MoCA “Attention” subdomain consists of brief, relatively simple items: digit span forward (1 point), digit span backward (1 point), and a vigilance task (1 point). These tasks primarily measure basic sustained attention and immediate auditory span. In contrast, the “Visuospatial/Executive” subdomain contains more complex tasks (trail-making, clock drawing, cube copying) that require sustained attention, working memory, cognitive flexibility, planning, and visuospatial integration simultaneously. A single error in any component can result in point loss, making these tasks more sensitive to the cumulative effects of anxiety-related resource depletion. Thus, the observed pattern is consistent with attentional control theory when task complexity is considered.

### 4.4 Mediation analysis

The mediation analysis suggests that depression statistically accounts for approximately 49.5% of the association between anxiety and cognitive function. The forward model (anxiety → depression → cognition) yielded an indirect effect of −0.153; the reverse model gave −0.148, indicating that direction cannot be inferred. Therefore, we interpret this as statistical mediation of association only.

Theoretically, this finding is consistent with the “anxiety-depression-cognition” cascade hypothesis, suggesting that anxiety and depression are not independent risk factors for cognitive impairment but rather form a potential pathway.

### 4.5 Age and sex differences

The age × depression interaction was marginally significant (*B* = −0.0018, *p* = 0.089), suggesting that the association between depression and cognition may strengthen with age. The sex × depression interaction was not significant (*B* = 0.045, *p* = 0.31). The descriptive differences in correlation coefficients between males and females should therefore be interpreted with caution and may reflect differences in variance or other patient characteristics rather than true effect modification.

### 4.6 Clinical implications

Our findings suggest that routine screening for both depression and anxiety should be implemented in neurology settings, as both are independently associated with cognitive impairment.

Our findings do not support clinical recommendations about the order of treating anxiety versus depression. Longitudinal and interventional studies are needed to establish causal relationships.

Elderly patients (≥75 years) warrant particular attention, as they show the strongest mood-cognition associations in this sample.

### 4.7 Limitations

Several limitations merit acknowledgment.

Additionally, because the forward (anxiety → depression → MoCA) and reverse (depression → anxiety → MoCA) mediation models yielded nearly identical results, our mediation findings cannot support directional claims. This is now explicitly stated in the mediation results and discussion sections.

First, the cross-sectional design precludes causal inference. While our mediation analysis is consistent with a pathway from anxiety to depression to cognition, the reverse mediation model showed comparable fit, and longitudinal data are needed to establish temporal precedence. All directional language has been replaced with associational language throughout the manuscript.

Second, the sample, while described as “neurology patients,” was predominantly composed of patients with ischemic cerebrovascular disease (90.9% in the reviewed subsample). This is a strength for internal validity but limits generalizability to other neurology populations (e.g., neurodegenerative or demyelinating diseases). We have revised the title and framing accordingly.

Third, while education data were incomplete (missing in 12.1% of patients), we included education as a covariate in all regression models to minimize confounding. The primary analysis used raw MoCA scores for maximum sample size, while education-adjusted scores were used in sensitivity analyses.

Fourth, the sample was predominantly cerebrovascular (90.9% in a diagnostic review of 318 patients), which limits generalizability to other neurology populations.

Fifth, we were unable to adjust for several potential confounders, including psychotropic medications (benzodiazepines, antidepressants), anticholinergic burden, vascular risk factors (hypertension, diabetes), and disease severity. Future studies incorporating these variables would help clarify the independent associations between mood and cognition.

Sixth, the inclusion criterion requiring independent questionnaire completion systematically excluded more severely cognitively impaired patients, potentially underestimating the true prevalence of cognitive impairment and attenuating observed mood-cognition associations through selection bias.

Seventh, the composite vulnerability index is a descriptive, non-validated metric; its ranking should be interpreted as exploratory rather than as an established construct.

### 4.8 Future directions

Longitudinal studies with repeated assessments of mood symptoms and cognitive function are needed to establish the temporal sequence of the anxiety-depression-cognition pathway. Neuroimaging studies could examine whether the observed associations are accounted for by structural brain changes, such as hippocampal volume or white matter hyperintensity burden (3). Clinical trials testing whether treatment of anxiety leads to improvement in depression and, subsequently, in cognitive function would provide the strongest evidence for causality. Finally, replication of the “U-shaped” pattern in independent cohorts is needed, along with studies examining the mechanisms underlying this phenomenon.

## 5 Conclusion

In this cross-sectional study of 667 patients with predominantly ischemic cerebrovascular disease, depression and anxiety were independently associated with cognitive impairment, with delayed recall being the most vulnerable domain. Mediation analysis revealed that 49.5% of the statistical association between anxiety and cognition was accounted for by depression, consistent with the hypothesized “anxiety-depression-cognition” pathway. Mood-cognition associations showed a marginal increase with age, and descriptive sex differences warrant further investigation. Prospective studies are needed to establish causality and to test whether treatment of mood symptoms improves cognitive outcomes.

## Data Availability

The raw data supporting the conclusions of this article will be made available by the authors, without undue reservation.

## References

[1] Blöchl M Nestler S. Long-term changes in depressive symptoms before and after stroke. Neurology. (2022) 99:e720–9. doi: 10.1212/WNL.0000000000200756, 35831179

[2] Loyal MS Numbers K Reppermund S Brodaty H Sachdev PS Mewton L . Longitudinal associations between late-life depression, cerebrovascular disease and cognition. J Affect Disord. (2025) 376:59–67. doi: 10.1016/j.jad.2025.01.147, 39892757

[3] Dintica CS Habes M Schreiner PJ Launer LJ Yaffe K. Trajectories in depressive symptoms and midlife brain health. Transl Psychiatry. (2024) 14:169. doi: 10.1038/s41398-024-02883-2, 38553474 PMC10980805

[4] Yin J John A Cadar D. Bidirectional associations of depressive symptoms and cognitive function over time. JAMA Netw Open. (2024) 7:e2416305. doi: 10.1001/jamanetworkopen.2024.16305, 38861255 PMC11167501

[5] Engedal K Gjøra L Benth JŠ Wagle J Rønqvist TK Selbæk G. The Montreal cognitive assessment: normative data from a large, population-based sample of cognitive healthy older adults in Norway-the HUNT study. J Alzheimer’s Dis. (2022) 86:589–99. doi: 10.3233/JAD-215442, 35094994

[6] Bugallo-Carrera C Dosil-Díaz C Pereiro AX Anido-Rifón L Gandoy-Crego M. Factors that indicate performance on the MoCA 7.3 in healthy adults over 50 years old. BMC Geriatr. (2024) 24:482. doi: 10.1186/s12877-024-05102-1, 38824525 PMC11144337

[7] Williams OA Demeyere N. Association of Depression and Anxiety with Cognitive Impairment 6 months after stroke. Neurology. (2021) 96:e1966–74. doi: 10.1212/WNL.0000000000011748, 33632803

[8] Perin S Lai J Pase M Bransby L Buckley R Yassi N . Elucidating the association between depression, anxiety, and cognition in middle-aged adults: application of dimensional and categorical approaches. J Affect Disord. (2022) 296:559–66. doi: 10.1016/j.jad.2021.10.007, 34648818

[9] Brown MJ Hill NL Haider MR. Age and gender disparities in depression and subjective cognitive decline-related outcomes. Aging Ment Health. (2022) 26:48–55. doi: 10.1080/13607863.2020.1861214, 33325263 PMC8206234

[10] Wu A Zhang J. Neuroinflammation, memory, and depression: new approaches to hippocampal neurogenesis. J Neuroinflammation. (2023) 20:283. doi: 10.1186/s12974-023-02964-x, 38012702 PMC10683283

[11] Zheng H Jia C. Gender differences in the association of depression trajectories with executive and memory functions: evidence from the longitudinal study of the survey of health, ageing and retirement in Europe (2004-2017). J Psychiatr Res. (2022) 149:177–84. doi: 10.1016/j.jpsychires.2022.03.007, 35278782

[12] Chang Z Cao X Zhang X Chen Y Wang L Zhang Y . Depressive symptoms and cognitive function trajectories in frail middle-aged and older adults: a longitudinal analysis based on the CHARLS cohort (2011-2018). Gen Hosp Psychiatry. (2025) 96:282–9. doi: 10.1016/j.genhosppsych.2025.08.006, 40812043

[13] Rock PL Roiser JP Riedel WJ Blackwell AD. Cognitive impairment in depression: a systematic review and meta-analysis. Psychol Med. (2014) 44:2029–40. doi: 10.1017/S0033291713002535, 24168753

[14] Ruthmann F Lo JW Mendyk-Bordet A-M Allart E Köhler S Klimkowicz-Mrowiec A . Prevalence of poststroke anxiety and its associations with global cognitive impairment: an individual participant data analysis. J Affect Disord. (2025) 369:1136–44. doi: 10.1016/j.jad.2024.10.099, 39447966

[15] Eysenck MW Derakshan N Santos R Calvo MG. Anxiety and cognitive performance: attentional control theory. Emotion. (2007) 7:336–53. doi: 10.1037/1528-3542.7.2.336, 17516812

